# Awareness of vitamin D deficiency among at-risk patients

**DOI:** 10.1186/1756-0500-5-17

**Published:** 2012-01-09

**Authors:** Esubalew Alemu, Robert Varnam

**Affiliations:** 1 Lancashire Teaching Hospitals, Sharoe Green Lane, Preston PR2 9HT, UK; 2 The Robert Darbishire Practice,Rusholme Health Centre, Walmer Street, Rusholme, Manchester M14 5NP, UK

## Abstract

**Background:**

Vitamin D deficiency is a significant problem for a growing proportion of the UK population. Individuals with dark or covered skin are at particularly high risk due to ethno-cultural, environmental and genetic factors. We assessed the level of awareness of vitamin D deficiency among at-risk patients in order to identify groups most in need of education.

**Findings:**

A cross-sectional survey using a piloted questionnaire was conducted among consecutive at-risk patients without a diagnosis of Vitamin D deficiency arriving at a large inner city general practice in the North West of England over a five day period. The survey was completed by 221 patients. The mean age was 35 years. 28% of them (n = 61) had never heard about vitamin D. Older patients (p = 0.003) were less likely to have heard about vitamin D. 54% of participants were unaware of the commonest symptoms of vitamin D deficiency. 34% did not expose their skin other than their face in the last one year, and 11% did not include vitamin D rich foods in their diet.

**Conclusion:**

The majority of at-risk patients are aware of vitamin D; nevertheless, there is a significant lack of knowledge among older people, who have higher morbidity. A programme of targeted education of the at-risk population is recommended.

## Background

Understanding of the role of vitamin D has been evolving since its discovery in the early 20th century from being a simple vitamin to a steroid pro-hormone. It has been recognised to be involved in various immune functions as well as bone and muscle development [1]. Vitamin D deficiency has been reported to be linked to depression, [2] autism, [3] type 1 diabetes, [4] Syndrome X, [5] as well as chronic widespread muscle and bone pain [6]. In infancy, it is associated with Rickets and hypocalcaemic fits [7,8].

On the other hand, the current evidence shows that wearing concealing clothes is associated with Vitamin D deficiency irrespective of race or strength of solar radiation [9-11]. Other studies reported higher incidence of Vitamin D deficiency among dark skinned patients due to decreased endogenous Vitamin D synthesis coupled with ethno-cultural as well as environmental factors [12-16]. Further, in the UK, a study of multi-ethnic patients from Birmingham revealed vitamin D deficiency prevalence of 1 in 4 Afro-Caribbeans and 1 in 3 Asians compared to 1 in 8 Caucasians [17].

Moreover, studies from Tunisia, New Zealand and the United States reported insufficient Vitamin D status in 47.6%, 54% and 100% of their participants respectively [18-20]. Other researchers documented that the prevalence of serum 25(OH)D levels of < 25 nmol/l was 9.3 times higher in Sri Lankans living in Norway compared with those in Sri Lanka [21]. Therefore, this data suggest that migration to northern latitudes, where there is lesser sun light, confers increased risk of developing vitamin D deficiency. Hence, ethno-cultural, environmental and genetic factors appear to increase the risk of Vitamin D deficiency among the population who cover their skin or have dark skin.

As regards awareness of patients, Kung et al. conducted a telephone interview of randomly selected Chinese women in Hong Kong about vitamin D knowledge and behaviour related to sunlight. The survey showed that among 547 participants, 62.3% did not like being exposed in the sun and the majority had heard about vitamin D despite low level of awareness of sources and role of vitamin D [22]. Likewise, Brand et al. conducted a qualitative exploratory study of 34 immigrants in Australia and reported the existence of limitations, variations between men and women as well as age differences in knowing about vitamin D deficiency [23]. Nevertheless, this study focused on the East Africans; therefore, it cannot be generalised for the rest of dark skinned community who could have different cultural norms. Thus, further studies incorporating wider range of dark skinned participants appear necessary.

In the UK, particularly in inner city areas, a significant minority of the population are at high risk of Vitamin D deficiency, as a result of latitude, dark skin and the wearing of dress such as the hijab and burka. In Rusholme, inner city part of Manchester, approximately, 33.7% of its population (n = 16,172) are from ethnic minorities with dark skin whilst about 47.7% (n = 2,927) of these being female who are more likely to cover up [24].

Without adequate appreciation of their at-risk status, and knowledge about lifestyle modifications which can prevent Vitamin D deficiency, this portion of the population are likely to continue to suffer morbidity from musculoskeletal pain and an increased risk of Rickets and infant fits.

## Aims

To ascertain the level of knowledge about Vitamin D deficiency in at risk people in an inner city area of the UK.

## Methods

A face-to-face survey was conducted of a sample of patients judged to be at risk of Vitamin D deficiency by virtue of their skin colour or clothing. This was done as part of a review of patient education for patients diagnosed with Vitamin D deficiency at an inner city GP practice in Manchester. This is an audit conducted following an approval by the clinical governance lead of the practice; hence, no external ethical approval was needed.

A survey tool was developed with reference to the literature on common risk factors for deficiency and measures of patient awareness and belief likely to influence relevant aspects of lifestyle. The survey thus assessed patients' ability to avoid Vitamin D deficiency. Its face validity was refined in a two stage pilot involving face to face interviews with 43 patients, resulting in the removal of some items and the rewording of others. The initial and final tools are shown in Table 1 and Table 2. Additional file 1: contains the questionnaire used in this survey.

**Table 1:** This table lists the questions of the initial questionnaire

1.	Have you ever had vitamin D deficiency?
2.	Do you take vitamin D supplements?

3.	Do you include vitamin D rich foods such as milk, fish oil or eggs in your meal?

4.	Have you done sunbathing within the last year whenever possible with exposed face, arms or legs?

5.	Do you know that vitamin D deficiency causes tiredness, low mood and muscle and bone pain?

6.	Where did you hear about vitamin D deficiency?

7.	Which sex are you? (If you are male, please go to Q9)

8.	Are you pregnant or breast feeding?

9.	Age: up to 25, 26-50, 51-75, above 76

10.	Ethnicity:

**Table 2:** This table list the questions of the final questionnaire

1.	Have you ever heard of Vitamin D? (If you answered 'No', please go to Q9)
2.	Do you think vitamin D is important for your health?

3.	Have you ever been told that you have vitamin D deficiency (not enough vitamin D)?

4.	Do you take vitamin D supplements (tablets)?

5.	Do you include vitamin D rich foods such as milk, fish oil or eggs in your meal?

6.	Have you been out in the sun within the last year with exposed face, arms or legs whenever possible?

7.	Do you know that vitamin D deficiency causes tiredness, low mood as well as muscle and bone pain?

8.	Where did you hear about vitamin D?

9.	Which sex are you? (If you are male, please go to Q11)

10.	Are you pregnant or breast feeding?

11.	What is your age?

12.	How do you think we can make patients of this surgery know more about vitamin D?

Every registered patient visiting the practice over a five day period from 1st of June 2009 to 5th of June 2009 was approached in the waiting room if they were of dark-skinned ethnicity or wearing garments providing total or near total skin coverage. Participants were consented and given the option of completing the survey on their own or with assistance such as reading the questions.

A data matrix was produced from the completed questionnaires using the SPSS 16.0 software. Variables for each question on the questionnaire were defined and entered according to their identification number on the SPSS Data Editor and analysed. The Chi Square Test was applied for comparing proportions and the *T *Test for mean differences and *p *< 0.05 was considered as statistically significant. Also used in the analysis process was the Microsoft Office Excel 2003. The calculated numbers were rounded off to the nearest whole number.

## Findings

### Participants

A total of 363 patients over the age of 18 years were approached and 293 (81%) of them participated. Seventy-two (25%) were excluded because they already had a diagnosis of Vitamin D deficiency, leaving 221 responses for analysis.

The mean age of these was 35 with Ninety-five (43%) of the respondents being women. Male participants were significantly older than females (mean age 37 vs 32 respectively, *p *= 0.004).

Respondents' level of awareness of Vitamin D and their responses regarding lifestyle risk factors are summarised in Table 3.

**Table 3:** This shows the number (percentage) of participants matched into categories mentioned in the table

Details	Number (Percentage)	Remarks
Total patients approached	363 (100%)	

Participants	293 (81%)	

Diagnosed with vitamin D deficiency	72 (25%)	Excluded

Non diagnosed with vitamin D deficiency	221 (75%)	Included

Never heard about vitamin D	N = 61 (28%)	
	
Heard about vitamin D	N = 160 (72%)	

Know the symptoms of vitamin D deficiency?	YES: N = 74 (46%)	
		
	NO: N = 86 (54%)	

Consume milk, fish or eggs?	YES: N = 143 (89%)	
		
	NO: N = 17 (11%)	

Taking over-the-counter vitamin D supplements?	YES: N = 10 (6%)	Missing data = 1
		
	NO: N = 149 (94%)	

More than their face exposed in the sun?	YES: N = 105 (66%)	
		
	NO: N = 55 (34%)	

### Knowledge about vitamin D

One hundred-sixty participants (72%) had heard about Vitamin D, leaving 61 (28%) who had never heard about it prior to completing the survey. Greater proportion of men than women had not heard about Vitamin D (33% and 21% 'never heard' respectively). However, this is not statistically significant (*p *= 0.069). Those who had not heard about Vitamin D were significantly older (mean age 38 vs 33, *p *= 0.024).

### Symptoms

Eighty-six respondents (54%) were unaware of the symptoms of Vitamin D deficiency. 45% and 61% of women and men respectively were not aware of the association of the symptoms with Vitamin D deficiency (*p *= 0.057). There was no age difference among those who knew the symptoms and who did not know (mean age 33, *p *= 0.888).

### Diet

A hundred and forty three participants (89%) included Vitamin D rich foods such as milk, fish or eggs in their meals whereas 11% of them did not include any of these foods. 9% and 12% of females and males respectively did not consume these foods (*p *= 0.798). There was a small, non-significant difference in age between those who did and did not consume these foods (mean age 34 vs 30, *p *= 0.268).

### Over-the-counter supplements

Six percent (n = 10) of participants reported taking over-the-counter Vitamin D supplements. 7% of men and 5% of women were taking the supplements. This indicates that larger proportion of men than women take supplements, nevertheless, there was no statistical significance (*p *= 0.752). Again, a small, non-significant age difference was observed between those who were and were not taking supplements (mean age 35 vs 33 respectively, *p *= 0.706). On the other hand, 6% (n = 8) and 12% (n = 2) of those who were and were not consuming Vitamin D rich foods respectively were taking Vitamin D supplements in spite of lack of statistical significance (*p *= 0.290).

### Exposure in the sun

Fifty-five participants (34%) had not exposed more than their face to the sun within the last year. The percentage of women who were not exposed to the sun was greater than that of men (36% vs 33% respectively). Nonetheless, this did not have statistical significance (*p *= 0.740). Those exposed in the sun were slightly younger than unexposed ones (mean age 33 vs 34 respectively, *p *= 0.648). On the other hand, those who were not exposed in the sun were more likely to take supplements as 15% of those unexposed in the sun were taking Vitamin D supplements and only 2% of those exposed were on supplementation (*p *= 0.003).

### Sources of information

**Figure 1:**
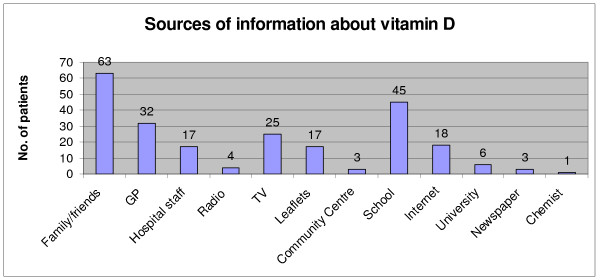
**This shows the number of patients in relation to the ways they heard about vitamin D**.

One hundred-sixty participants (72%) responded to the question regarding how they heard about Vitamin D. The results are presented in Figure 1. Family and friends were the most popular source cited by 63 participants and followed by school education [n = 45] and GP [n = 32]. Also mentioned were television [n = 25], hospital staff [n = 17], the internet [n = 18] and leaflets [n = 17].

**Figure 2:**
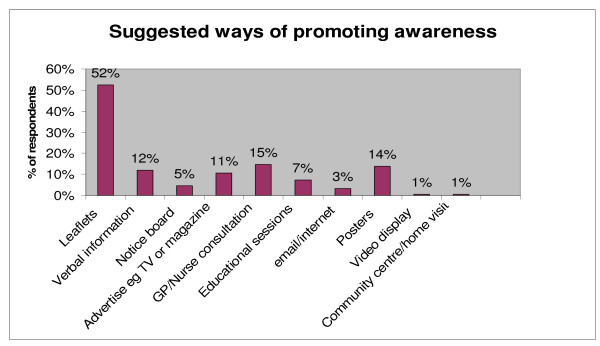
**This shows the percentage of participants in relation to their suggestions of ways of promoting awareness**.

### Suggested ways of promoting awareness

A hundred and twenty five respondents (57%) suggested ways of promoting awareness among the at-risk population. The results are presented in Figure 2. The most common suggestion was use of leaflets mentioned by 52% followed by GP/Nurse consultation (15%) and poster display (14%). Also suggested ways include providing verbal information (12%), advertising on media (11%) and organising educational sessions (7%).

## Discussion

The results identify a significant unmet need for education about vitamin D among people at risk of deficiency. Nearly a third of people at risk of deficiency by virtue of dark or covered skin had never heard of it whereas just over a third were getting almost no sun exposure and very few took appropriate supplements.

On the other hand, men and older people had particularly low levels of awareness of Vitamin D deficiency and its potential consequences. Gender influences on health related attitudes and behaviour are common findings [23,25]. The influence of age on Vitamin D awareness confirms findings of a survey among patients in Hong Kong [22] and highlights the inaccessibility and lack of perceived relevance of much health information to the older generation.

As regards this study, since the data was collected with a questionnaire mainly comprising of closed questions, the fact gathered is limited. Moreover, individuals who turned up at the practice may be different from those who did not in relevant ways. This was a small non-random sample of residents in one UK city, albeit with a high response rate. Hence, further research is sought in order to confirm the findings.

Fresh effort is recommended to address the needs of the at risk population for education about Vitamin D deficiency and ways of avoiding it. This should address both suggested lifestyle modifications and symptoms, which warrant consultation with the GP. A third of participants had received information about Vitamin D from family or friends. While the social connections of older at-risk people may differ from this group, community networks appear to be a promising vector for the dissemination of education about this important problem. The group with greatest need for education are also more likely than the general population to consult their GP, and this may also prove an important source of education.

Recommendations to change lifestyle factors in Vitamin D deficiency may not be popular, as other work has shown [26] and the lower levels of UVB radiation received from the sun at higher latitudes may significantly limit the benefit of sun exposure in the UK. The role of the GP in offering pharmacological Vitamin D to at risk patients may therefore be paramount.

## Conclusion

This study highlighted the existence of differences in awareness of Vitamin D deficiency among men and women whilst age playing a significant role. Thus, a concerted collaboration is required amongst the health care providers and others such as community members to promote the awareness and bridge this gap.

## Abbreviations

25(OH)D: 25-Hydroxyvitamin D; GP: General Practitioner.

## Competing interests

The authors declare that they have no competing interests.

## Authors' contributions

EA designed the questionnaire, approached the patients, collected the data, performed statistical analysis and drafted the manuscript.

RV supervised designing of the questionnaire, data collection and data analysis as well as editing and finalising the manuscript. All authors read and approved the final manuscript.

## Supplementary Material

Additional file 1 **Questionnaire used for data collection**. Click here for file
