# Supplementary material for: Awareness of vitamin D deficiency among at-risk patients
Source: BMC Res Notes. 2012 Jan 9;5:17. doi: 10.1186/1756-0500-5-17 (PMC3284868; doi:10.1186/1756-0500-5-17)
Supplement: Additional file 1 — Questionnaire used for data collection. [file 1756-0500-5-17-S1.DOC]

**VITAMIN D DEFICIENCY AWARENESS QUESTIONNAIRE**

The purpose of this questionnaire is to find out the awareness of our patients about Vitamin D deficiency and improve the medical care accordingly. Any information you give us is confidential and handled in the strictest manner.

Please tick your answer.

1. Have you ever heard of Vitamin D?

Yes No

***If you answered ‘No’, please go to Q9****.*

1. Do you think vitamin D is important for your health?

Yes No Do not know

1. Have you ever been told that you have vitamin D deficiency (not enough vitamin D)?

Yes No

1. Do you take vitamin D supplements (tablets)?

Yes No

1. Do you include vitamin D rich foods such as milk, fish oil or eggs in your meal?

Yes No

1. Have you been out in the sun within the last year with exposed face, arms or legs whenever possible?

Yes No

1. Do you know that vitamin D deficiency causes tiredness, low mood as well as muscle and bone pain?

Yes No

1. Where did you hear about vitamin D?

Family/friends GP Hospital Radio TV Leaflets Community centres School Internet Other:………………………

1. Which sex are you? Female Male

***If you are male, please go to Q11.***

1. Are you pregnant or breast feeding?

Yes No

1. What is your age?........................................................................................................
2. How do you think we can make patients of this surgery know more about vitamin D?

………………………………………………………………………………………………………………………………………………………………………………………………

……………………………………………………………………………………………..Thank you for completing this questionnaire!
